# Chemical Composition and Antimicrobial Activities of *Artemisia argyi* Lévl. et Vant Essential Oils Extracted by Simultaneous Distillation-Extraction, Subcritical Extraction and Hydrodistillation

**DOI:** 10.3390/molecules24030483

**Published:** 2019-01-29

**Authors:** Xiao Guan, Depeng Ge, Sen Li, Kai Huang, Jing Liu, Fan Li

**Affiliations:** 1 School of Medical Instruments and Food Engineering, University of Shanghai for Science and Technology, Shanghai 200093, China; gedepeng@hotmail.com (D.G.); lisen_1027@126.com (S.L.); hkjn1990@163.com (K.H.); hkshlg2018@163.com (F.L.); 2 College of Information Engineering, Shanghai Maritime University, Shanghai 200135, China; jingliu@shmtu.edu.cn

**Keywords:** *Artemisia argyi*, simultaneous distillation extraction, essential oil, antimicrobial activity

## Abstract

*Artemisia argyi* Lévl. et Vant essential oil could be used as a good antimicrobial flavouring agent and applied in the food industry. In this study, three methods, including simultaneous distillation-extraction (SDE), subcritical extraction and hydrodistillation, were applied to extract *A. argyi* essential oil. Compared with subcritical extraction (1%) and hydrodistillation (0.5%), SDE gave a higher yield (1.2%). Components of the essential oils were analysed with gas chromatography-mass spectrometry (GC-MS), and the most abundant ingredients were caryophyllene oxide, neointermedeol, borneol, α-thujone and β-caryophyllene. These five components accounted for 82.93%, 40.90% and 40.33% for SDE, subcritical extraction, and hydrodistillation, respectively. Based on agar disc diffusion and minimum inhibitory concentration (MIC) assays, SDE oil showed a significant inhibitory effect towards *Listeria monocytogenes*, *Escherichia coli*, *Proteus vulgaris*, *Salmonella enteritidis* and *Aspergillus niger*. Furthermore, electron microscope observations (SEM) confirmed that SDE oil could obviously deform cell morphology and destroy the structure of cell walls. Performances showed that SDE was a promising process for extracting *A. argyi* essential oil with both high yield and antimicrobial activity.

## 1. Introduction

*Artemisia argyi*, known as Chinese mugwort, is an herbaceous perennial plant with creeping rhizomes. *A. argyi* is the most popular plant in China and eastern Asia, and its leaves are used as a Traditional Chinese Medicine (TMC). *Artemisia* species are important medical plants which have aroused interest for their biological and chemical diversities [1]. Known for a long time for the treatment of diseases such as asthma, inflammation, hepatitis and infections by bacteria, fungi or viruses [2], *A. capillaris herba* has officially been listed in the Chinese Pharmacopoeia and applied in the treatment of epidemic hepatitis [3]. In addition, in Europe, the use of *A. mexicana* to stimulate the appetite and to aid digestion is allowed [4].

The main pharmacological active compound in *A. argyi* is the essential oil and the biological activities of the extracted essential oil have been investigated. For example, it has been reported that *A. argyi* essential oil showed anti-histaminic effects and antifungal activity [5]. Moreover, *A. argyi* volatile oil had strong antibacterial effects against *Staphylococcus aureus*, *Escherichia coli* and *Salmonella enteritidis* [6]. Gas chromatography-mass spectrometry (GC-MS) has been proved to be an useful method for the determination of the different essential oil components. Recently, the composition of essential oil extracted from leaves and flowers of *A. argui* has been reported [7], and the major components were sesquiterpenes, monoterpenes, alcohols, ketones, aromatic compounds esters and ethers, etc. Based on the appealing aromatic properties, *A. argui* essential oil could play a significant role in food safety and preservation [8]. At present, most preservatives used by the food industry are artificial additives such as nitrates, sulphur dioxide and benzoates [9]. Natural compounds may have great commercial value in the food industry, however their applications are currently limited owing to their high cost. Hence, an effective and low-cost preparation method is needed.

Classical methods of extraction, such as solid-liquid extraction with organic solvents, are used for the extraction of lipid fractions and essential oils from aromatic plants. These solvents provide good recovery of oil and other compounds, but they have certain drawbacks such as potential human and environmental toxicity. Compared with the classical methods, hydrodistillation has been the most common method used to extract the essential oils from plants. Although this method still has drawbacks such as long extraction times and high energy use, hydrodistillation is the simplest and most practical method [10,11]. Recently, there has been great interest in subcritical extraction for its mild operational temperature, no solvent residues and better nutrition retention. Because subcritical solvents have the advantages of high density, high diffusively and low viscosity, the subcritical extraction method has been widely applied in lipid and essential oil extraction [12]. Furthermore, it is reported that simultaneous distillation-extraction (SDE) could be also used in essential oil extraction. This one-step extraction technique is less time consuming and allows a greater reduction of solvent volumes due to the continuous recycling. Under certain conditions a higher yield and richer ingredients could be achieved and the extracts obtained by SDE are free from non-volatile materials such as cuticular waxes and chlorophylls [13,14]. Recently, our laboratory has focused on searching for valuable plant essential oils which could be applied in the food safety and preservation. Up to now, *A. argyi* essential oils extracted by simultaneous distillation have never been reported. In this study, we examined different methods, including SDE, subcritial extraction and hydro-distillation, for *A. argyi* essential oil extraction and characterized the corresponding compositions. The aim of this study was to evaluate the effects of these methods on the yield and the biological characteristics of the essential oil, and to analyze its inhibitory effects on microorganisms that cause vegetable, fruit and other food losses.

## 2. Results and Discussion

### 2.1. Extraction Yields of A. argyi Essential Oils

*A. argyi* essential oils were extracted with hydrodistillation, subcritical extraction and SDE respectively. Their extraction time, yields and colors were evaluated. As shown in Table 1, SDE took the shortest time (180 min) and gave the highest yield (1.2%), followed by subcritical extraction (1%) and hydrodistillation (0.5%). The yields of these extracted oils were higher than the reported ones, which were around 0.20% to 0.26% [15]. Furthermore, SDE cost 1 h less than hydrodistillation, and 2 h less than subcritical extraction. Compared with the other studies, SDE is a feasible method which offers yield and processing time advantages. 

### 2.2. Compositions of A. argyi Essential Oils

The identified components of *A. argyi* essential oils obtained from the three different extraction methods and their concentrations are shown in Table 2.

**Figure 1:**
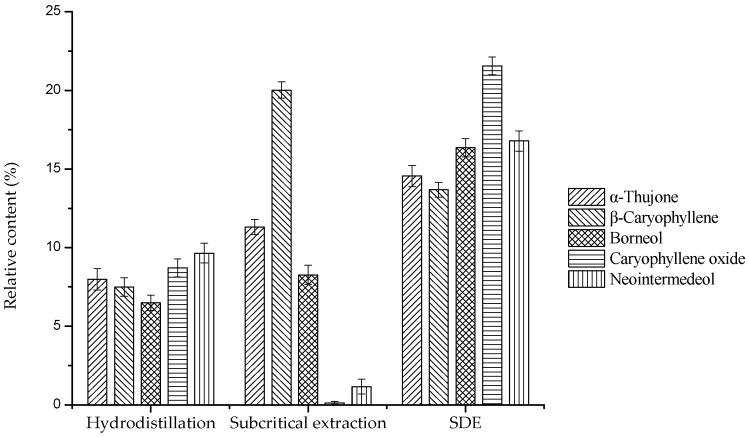
The major chemical components of *Artemisia argyi* essential oils (in percentages, average of a duplicate assay).

The contents of compounds varied markedly along with the extraction method. As shown in Table 2, a total of 65, 62 and nine compounds were identified for the essential oil extracted with hydrodistillation, subcritical extraction and SDE, accounting for the 86.318%, 94.496% and 99.997% of total oil, respectively. Different from SDE oil, subcritical extraction oil and hydrodistillation oil were dominated by the monoterpene fractions with 62.479% and 48.021%, respectively. However, the total content of monoterpene fractions in SDE oil was 45.49% and no monoterpene hydrocarbon fractions were determined. On the other hand, SDE oil was dominated by the sesquiterpene fractions, and large amount of the oxygenated sesquiterpene (40.82%) was found. Although different extraction methods produced essential oils with various chemical compositions, there were five major compounds in common, including caryophyllene oxide, neointermedeol, borneol, *α*-thujone and *β*-caryophyllene. As shown in Figure 1, the total of these five compounds accounted for around 82.93%, 40.90% and 40.33% for SDE, subcritical extraction and hydrodistillation, respectively. Similarly, it is well-documented that caryophyllene oxide, neointermedeol, borneol, *α*-thujone and *β*-caryophyllene had the highest contents [16]. Mevy et al. have proved that these components exhibited antioxidant, antibacterial and other biological activities [17]. Additionally, compared with the other two methods, the contents of these common ingredients in SDE essential oil was obviously higher. Usually, traditional essential oil extractions are time consuming and high temperature needed. These methods might lose some volatiles and significantly degrade unsaturated compounds or esters [18]. Similarly, SDE would also result in severe losses of volatile materials, because oil-containing hexane should be subsequently removed by rotary evaporation.

### 2.3. Antimicrobial Activities of A. argyi Essential Oils

**Figure 2:**
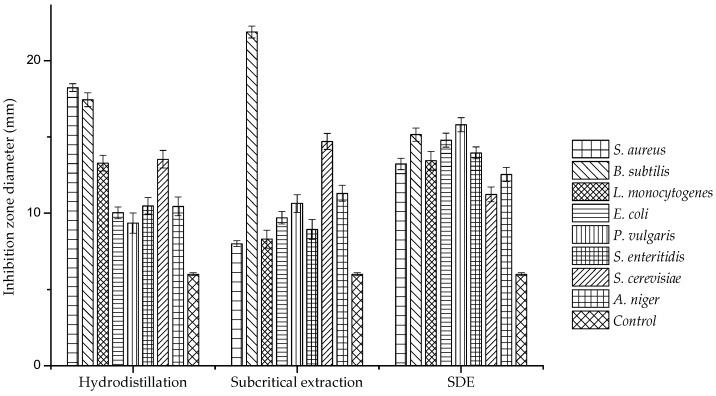
Inhibition zones of *Artemisia argyi* essential oils against various microorganisms.

The antimicrobial activities of *A. argyi* essential oils determined by the agar disc diffusion assay are listed in Table 3. In general, Gram-positive pathogens were much more sensitive to *A. argyi* essential oil than fungi and Gram-negative pathogens. *Bacillus subtilis* was the most sensitive bacterium, with an inhibition diameter longer than 15 mm and *Escherichia coli* was the most inhibited Gram-negative bacterium with an inhibition diameter longer than 9.7 mm. As shown in Figure 2, hydrodistillation oil showed strong inhibition against *S. aureus* and *B. subtilis*, whereas subcritical extraction oil showed significant inhibition against *B. subtilis* and *S. cerevisiae*, with inhibition zone diameters of 21.88 and 14.7 mm, respectively.

**Figure 3:**
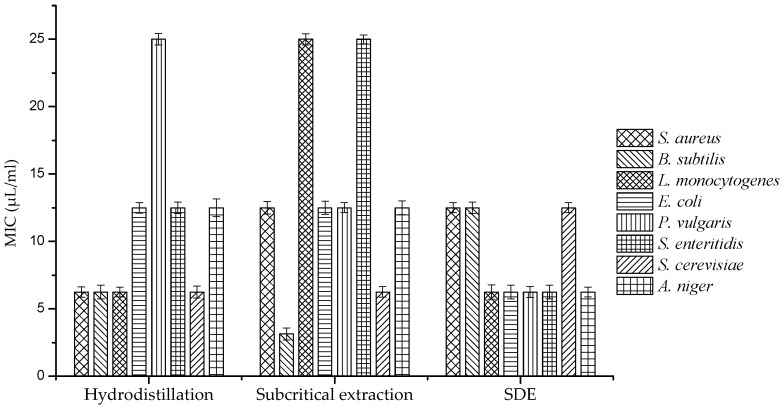
MICs of *Artemisia argyi* essential oils against various microorganisms.

Interestingly, SDE oil showed a broad spectrum of antimicrobial activity with a 13 mm diameter on average. Furthermore, MICs of different *A. argyi* essential oils against eight microbes were also tested (listed in Table 4 and shown in Figure 3). MICs of SDE oil against most microorganisms were 6.25 μL/mL, peaking at 12.5 μL/mL. In contrast, MICs of hydrodistilled and subcritical extracts were mostly higher than 12.5 μL/mL and several values even reached 25 μL/mL. As the results above indicate, SDE oil showed a higher antimicrobial activity than the other two oils, which suggested that SDE oil could be a good candidate for an antimicrobial agent. Danh et al. reported that the antimicrobial activity of *A. argyi* essential oil was predominantly controlled by the amount of active compound with high diffusivity in agar medium [19]. During hydrodistillation and subcritical extraction, compounds could be hardly diffused, and those with high hydrophobicities exerted little influence on the antimicrobial activities of resultant oils [20]. The antimicrobial activities of essential oils were apparently related to the large amount of caryophyllene oxide, neointermedeol, borneol, *α*-thujone and *β*-caryophyllene. The activity of SDE oil was superior to those of subcritical and hydrodistilled extracts, which displayed lower MICs and larger inhibition zones. Compared with SDE, hydrodistillation needs large quantity of water and higher temperature, which would cause hydrolysis reaction and damage the active compound. Subcritical extraction would bring some miscellaneous ingredients such as wax and pigment. These ingredients might be the reason for the less inhibitory effect of essential oil extracted by subcritical extraction [12]. On the other hand, the highest antimicrobial activity of essential oil extracted by SDE might also be related with *n*-hexane which has the appropriate polarity to accumulate active antimicrobial ingredients. 

**Figure 4:**
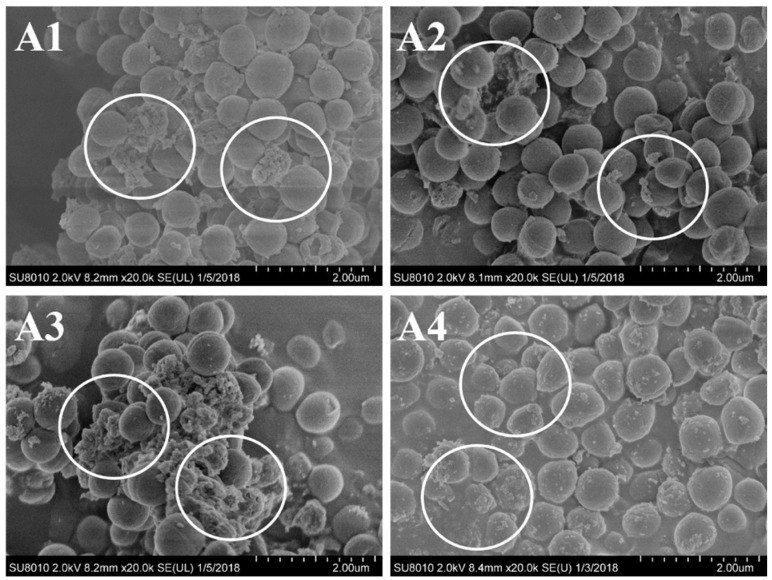
SEM images of *S. aureus* treated with *Artemisia argyi* essential oils obtained by hydrodistillation (**A1**), subcritical extraction (**A2**) and SDE (**A3**) at the MICs, respectively. And (**A4**) is control sample.

**Figure 5:**
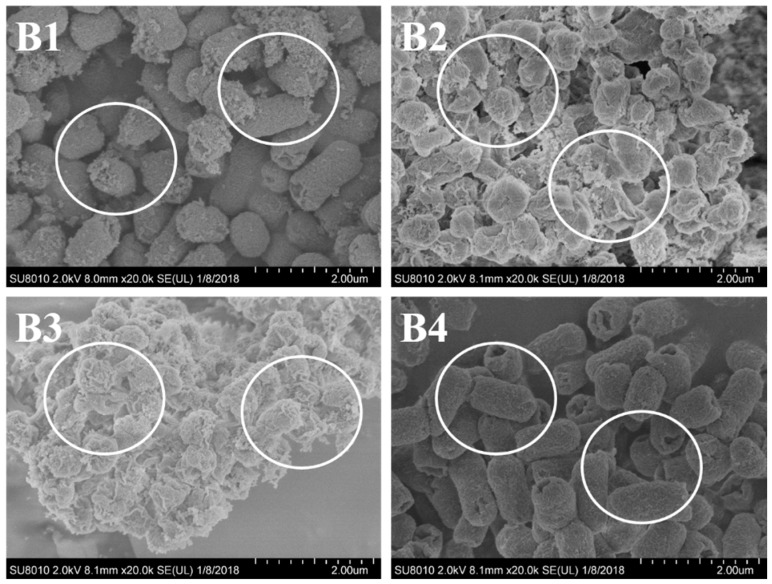
SEM images of *E. coli* treated with *Artemisia argyi* essential oils obtained by hydrodistillation (**B1**), subcritical extraction (**B2**) and SDE (**B3**) at the MICs, respectively. And (**B4**) is control sample.

Taking both of the results from MICs and inhibition zone diameter into consideration, *S. aureus* and *E. coli* were the appropriate target bacteria with higher sensitivity. To further investigate the antimicrobial activity of the extracted essential oils, SEM analysis was performed using the two selected sensitive bacteria. As shown in Figure 4 and Figure 5, the images directly exhibited the detrimental inhibitory effect of the essential oil from *A. argyi* against the tested bacteria. It could be found that, for the most part, control cells were intact and showed a smooth surface or weakly damaged (Figure 4(A4) and Figure 5(B4)). In contrast, bacterial cells treated with the *A. argyi* essential oils were subjected to considerable damage (Figure 4(A1–A3) and Figure 5(B1–B3)). Similar results have been reported by Diao et al. [21]. These results revealed that the active compounds from essential oil might bind to the cell surface and then penetrate into the target sites, which could destroy the structure of cell walls. It should be noticed that, as shown in Figure 4 and Figure 5, the cell walls changed much to different extents after treated with the essential oil, while SDE oil showed the strongest inhibitory effect against the strains. 

## 3. Materials and Methods

### 3.1. Materials

Leaves of *A. argyi* Lévl. et Vant were purchased from an *A. argyi* farm (Qichun, China). The plant was identified by ProfessorXu Fei, from the School of Food Science and Technology, University of Shanghai for Science and Technology. The leaves were ground into powders and stored at 4 °C.

### 3.2. Strains

The antimicrobial activities of essential oil samples were tested against eight different microorganisms including three Gram-positive bacteria *Staphylococcus aureus* (ATCC 6538), *Bacillus subtilis* (CMCC B63501) and *Listeria monocytogenes* (ATCC 19115), three Gram-negative bacteria *Escherichia coli* (ATCC 12900), *Proteusbacillus vulgaris* (CMCC B49027) and *Salmonella enteritidis* (ATCC 13076), as well as two fungi: *Saccharomyces cerevisiae* (ATCC 9763) and *Aspergillus niger* (ATCC 6275). These strains were obtained from the School of Medical Instrument and Food Engineering, University of Shanghai for Science and Technology (Shanghai, China), and stored in glass ampoules at −80 °C prior to use. In this test, nutrient broth (NB), potato dextrose agar (PDA), lauryl sulfate tryptose broth (LSTB), Czapek dox agar (CDA), yeast extract dextrose chloramphenicol agar (YEDCA) and modified Czapek dox broth (MCDB) medium were used as culture media, and there were purchased from Sinopharm Chemical Reagent Co., Ltd. (Shanghai, China).

### 3.3. Extraction of Essential Oils

#### 3.3.1. Hydrodistillation

Ground *A. argyi* samples (100 g) mixed with 1500 mL deionized water were hydrodistilled in a glass Clevenger-type apparatus for 4 h. Details of the procedures were followed as the previous report [22]. Extracted oil was weighed and stored at 4 °C, and the experiment was repeated three times. 

#### 3.3.2. Subcritical Extraction

**Figure 6:**
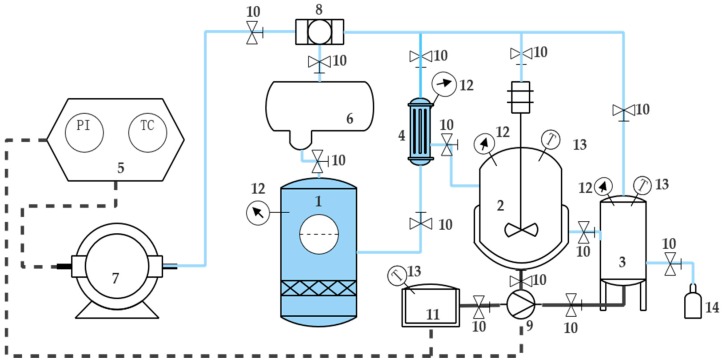
Schematic diagram of subcritical extraction experimental apparatus. Parts of the apparatus are numbered as follows: 1. Butane solvent storage pot; 2. Extraction agent; 3. Knockout drum; 4. Solvent bottle; 5. Control panel; 6. Condenser; 7. Compressor; 8. Filter; 9. Water pump; 10. Spherical value; 11. Hot water tank; 12. Piezometer; 13. Thermometer; 14. Collection bottle.

The subcritical extraction system (shown in Figure 6) was provided by Henan Subcritical Biological Technology Co., Ltd. (Henan, China). In this study, 100 g ground *A. argyi* sample was loaded into the extraction cell. Butane from the storage pot (No. 1) was passed to refrigerator to be cooled and liquefied. Then liquefied butane was pressurized by the compressor (No. 7). Subsequently, compressed butane was passed into the solvent bottle (No. 4) and then transferred to the extraction agent (No. 2). In the shell-and-tube heat exchanger, water was circulated in its shell with constant temperature, providing the required temperature for extraction. The operation time was determined for all experiments by closing the extraction agent (No. 2) valve for about 5 h. The obtained essential oil was collected carefully from knockout drum (No. 3). Because the essential oil was sensitive to light and heat, it was carefully weighed and kept in a sealed murky vial in an ice box before analysis. According to the reported method [23], the experiment was repeated three times.

#### 3.3.3. Simultaneous Distillation-Extraction (SDE)

**Figure 7:**
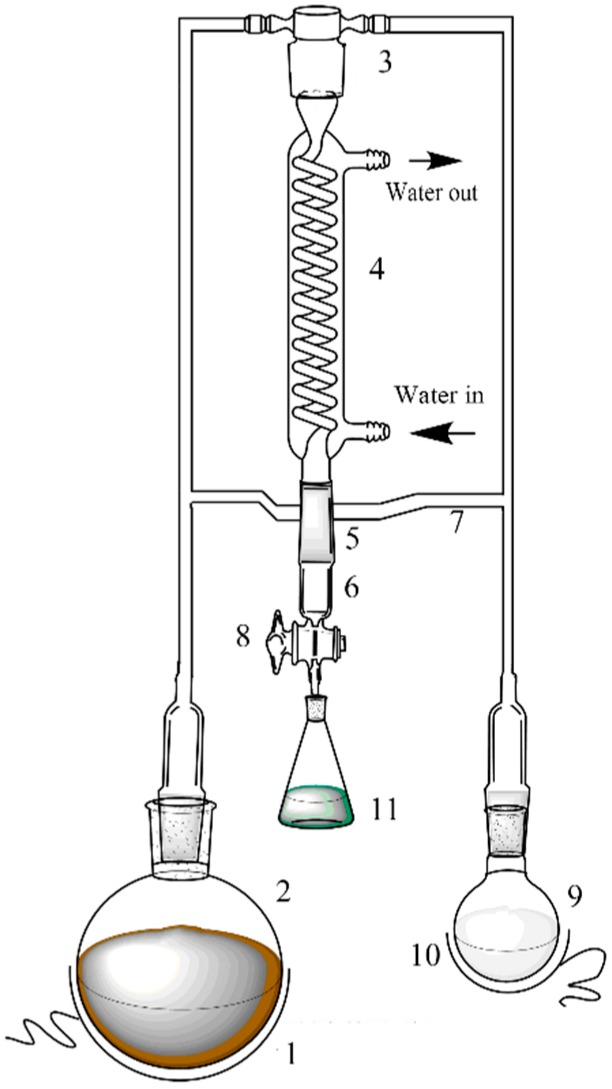
Schematic diagram of SDE experimental apparatus. Parts of the apparatus are numbered as follows: 1. Thermostat; 2. extraction agent (water); 3. mixing zone of water steam and n-hexane steam; 4. condenser; 5. extraction cell; 6. cooling jacket; 7. phase separation tube; 8. valve; 9. reaction flask containing n-hexane; 10. thermostat; 11. collection bottle.

Simultaneous distillation-extraction of *A. argyi* essential oil was performed with a specially designed equipment (shown in Figure 7). Ground *A. argyi* sample (100 g) was loaded into the flask (extraction agent, No. 2) with 1500 mL deionized water, and coupled with another flask (No. 9) which contained 30 mL hexane. The operating temperatures of thermostats No. 1 and 10 were set at 100 °C and 45 °C respectively. The extraction system was performed for 3 h. Afterwards, the valve (No. 8) was turned on, and the continuously condensed distillate was collected in the receiver (No. 11). The essential oil and hexane were separated by a rotary evaporator. Subsequently, the extract was weighed and stored at 4 °C. On the basis of the reported method [24], the experiment was repeated three times.

#### 3.3.4. Extraction Yield Calculation

The extraction yields of *A. argyi* essential oil were calculated as follows:

(1) $$\mathrm{Extraction}\;\mathrm{yield}=\frac{\mathrm{Weight}\;\mathrm{of}\;\mathrm{essential}\;\mathrm{oil}}{\mathrm{Weight}\;\mathrm{of}\;\mathrm{ground}\;\mathrm{samples}}\times 100$$

### 3.4. Chemical Analysis of Essential Oil

The GC-MS system (Agilent, Little Falls, DE, USA) was composed on a 5975 mass selective detector and a 6890 GC. The GC capillary column was an Agilent HP-5MS (30 m × 0.25 mm × 0.25 μm). The injector and detector temperatures were 220 and 260 °C, respectively. The oven temperature was held at 45 °C for 5 min, increased to 250 °C with a flow rate of 3 °C /min, and then held for 10 min. A 1 μL aliquot of sample was injected in the split mode with a ratio of 1:10. Helium was used as the carrier gas with a flow rate of 1 mL/min. 

Samples were analyzed by GC-MS and the data were analyzed using the MetAlign^TM^. Software (version 3.0, Wageningen University, 2011, http://www.MetAlign.nl). In this untargeted approach, the software was used to align and compare full scan GC-MS chromatograms and assess possible difference between samples. Raw data from GC-MS chromatograms were imported into MetAlign^TM^, and baseline correction, denoising and smoothing of the data were carried out. This was achieved by setting parameters in the MetAlign^TM^ interface, where the minimum peak threshold to eliminate noise that would interfere with peak alignment, was determined. Spectral alignment was performed using the rough mode, where ion fragments originating from chromatographic peaks with corresponding retention times were aligned across all samples.

### 3.5. Detection of Antimicrobial Activity by Agar Disc Diffusion Assay

The agar disc diffusion assay was carried out to evaluate the antimicrobial activities of the essential oils with different extraction methods. Briefly, 100 μL aliquot of microbial inocula in the exponential growth phase (cell concentration of 10^5^–10^6^ CFU/mL) were spread over the plate surfaces. The three essential oils were dissolved in 1% dimethyl sulfoxide to 25 µL/mL. Four sterile test discs (Ф = 6 mm) were placed onto each agar plate and then injected with 10 μL essential oil individually. Negative control was prepared using 1% dimethyl sulfoxide and the antibiotic amoxicillin was used as positive control at the concentration 50 μg/mL. The plates were incubated for 24 h at 37 °C for bacteria and for 48 h at 30 °C for yeasts. Full diameter of the zone was regarded as the inhibition zone diameter, which was read by eye and measured by ruler. The tests were performed in four replicates for each sample.

### 3.6. Measurement of Minimum Inhibitory Concentration (MIC)

The three essential oils were first dissolved in 1% dimethyl sulfoxide and diluted to 50.000, 25.000, 12.500, 6.250, 3.125, and 1.562 µL/mL respectively. Then 1 mL of each diluted sample was mixed with 14 mL of PDA culture medium and poured into a sterile petri dish. Subsequently, 1 mL culture medium was inoculated onto the potato dextrose agar plates and incubated for 48 h at 28 °C. The growth of the strains was monitored and the assay was performed in triplicate. The MIC was defined as the lowest concentration of the essential oil at which the microorganism tested did not demonstrated visible on the plates [25].

### 3.7. Scanning Electron Microscope Observations

SEM studies were carried out to observe the morphological changes of bacteria, which were treated with MIC value of *A. argyi* essential oil. Cells were harvested by centrifugation for 15 min at 5000 r/min, and washed three times with 0.1 M phosphate buffer solution (PBS, PH 7.4). The treatment of bacterial cells and observations of SEM was followed by the reported study [26]. All the samples were coated with gold in a sputter coater, followed by microscopic examinations using a SEM system (S-3000H; Hitachi Ltd., Tokyo, Japan).

### 3.8. Statistical Analysis

ANOVA was performed using Statistical Package for Social Sciences (SPASS 20.0, SPASS Inc., Carlsbad, CA, USA) for comparing mean values at *p* < 0.5.

## 4. Conclusions

*A. argyi* essential oil has aroused attention due to its biological activities. In this study, three methods were applied to extract the essential oil from *A. argyi* leaves. The effects of each method on the yields, chemical composition and bioactivity were investigated. The yield of SDE extract was nearly 1.2%, which was comparable to the subcritical extract (1%), while much higher than the hydrodstilled oil (0.5%). Although the chemical compounds of the SDE oil was much less than hydrodistilled oil and subcritical extract oil, the major compounds including caryophyllene oxide, neointermedeol, borneol, *α*-thujone and *β*-caryophyllene of SDE oil accounted for 82.93%, which was higher than subcritical extraction and hydrodistillation. The antimicrobial activities of these oils were estimated using agar disc diffusion and MIC assays. They showed that SDE oil had a good antimicrobial activity, and the activity against tested microorganisms like *L. monocytogenes*, *E. coli*, *P. vulgaris*, *S. enteritidis* and *A. niger* was higher or equal to those of other two extracts. Furthermore, SEM results showed that *A. argyi* essential oil could destroy the structure of cell walls and the SDE oil had the strongest inhibitory effect against the tested strains. All these results above suggested that SDE *A. argyi* essential oil had the advantages in yield, bioactive ingredients and antimicrobial activities, which qualified that SDE oil could be a good preservative candidate for the food industry.

## Figures and Tables

**Table 1:** Times and yields of *Artemisia argyi* essential oils obtained by three different extraction methods.

Extraction Method	Extraction Time (min)	Yield (% Dry Weight)	Colour
Hydrodistillation	240	0.50%	Dark green
Subcritical extraction	300	1%	Yellow
SDE	180	1.20%	Pale yellow

**Table 2:** Chemical compositions (in percent) of *Artemisia argyi* essential oils obtained by three different methods.

Compounds	CAS	Relative Content (%)		
		Hydrodistillation	Subcritical Extraction	SDE
**Monoterpene hydrocarbons**		**0.87**	**1.61**	**14.55**
*γ*-Terpinene	000099-85-4	0.247	0.468	—
*o*-Cymene	000527-84-4	0.315	0.567	—
Terpinolene	000586-62-9	0.079	0.154	—
*α*-Thujene	002867-05-2	0.233	0.42	14.551
**Oxygenated monoterpenes**		**47.38**	**61.29**	**45.49**
2,5,5-Trimethyl-2,6-heptadien-4-one	000512-37-8	0.048	0.569	—
Yomogi alcohol	026127-98-0	0.535	2.877	—
*α*-Thujone	000546-80-5	7.989	11.312	14.551
*β*-Thujone	000471-15-8	1.916	1.928	—
*trans*-Sabinene hydrate	017699-16-0	1.199	0.653	—
2,2,4-Trimethyl-3-cyclohexene-1-carbaldehyde	001726-47-2	—	0.15	—
(+)-2-Bornanone	000464-49-3	3.896	7.253	10.022
*trans*-Pinocamphone	000547-60-4	0.179	0.216	—
Umbellulone	024545-81-1	0.407	0.123	—
*cis*-2-Menthenol	029803-82-5	1.614	—	—
*trans*-Chrysanthenyl acetate	054324-99-1	—	0.156	—
Bornyl acetate	000076-49-3	0.24	0.296	—
Dill ether	070786-44-6	0.059	0.087	—
l-Terpinen-4-ol	000562-74-3	4.441	0.233	—
*trans*-Dihydrocarvone	005948-04-9	0.252	—	—
Benihinal	000564-94-3	0.275	0.464	—
*trans*-2,8-*p*-Mentha-dien-1-ol	007212-40-0	0.102	0.173	—
*cis*-2-Menthenol	029803-82-5	—	2.351	—
(−)-*trans*-Pinocarveol	000547-61-5	0.349	0.835	—
Verbenol	000473-67-6	0.209	0.153	1.827
Borneol	000507-70-0	6.482	8.273	16.356
*cis*-Sabinol	003310-02-9	5.505	1.747	—
Verbenone	000080-57-9	—	0.913	—
Isothujol	000513-23-5	1.193	—	—
*α*-Terpineol	000098-55-5	3.617	4.119	—
Piperitone	000089-81-6	0.422	0.49	—
*α*-Phellandren-8-ol	001686-20-0	—	0.932	—
*cis*-Chrysanthenol	055722-60-6	2.529	4.209	2.738
*trans*-Piperitol	016721-39-4	0.602	1.062	—
Myrtenol	000515-00-4	—	0.369	—
*trans*-*p*-Mentha-1(7),8-dien-2-ol	021391-84-4	0.436	0.911	—
4-Isopropyl-1,5-cyclohexadiene-1-methanol	019876-45-0	0.123	0.063	—
Dihydrocarveol	000619-01-2	0.614	0.468	—
*cis*-Carveol	001197-06-4	1.404	2.997	—
*p*-Cymene-8-ol	001197-01-9	0.425	0.707	—
*trans*-Shisool	022451-48-5	0.01	—	—
*β*-Ionone	000079-77-6	0.028	4.13	—
*p*-Isopropylbenzyl alcohol	000536-60-7	0.17	0.072	—
Thymol	000089-83-8	0.108	—	—
**Sesquiterpene hydrocarbons**		**12.702**	**26.846**	**13.687**
*α*-Cubebene	017699-14-8	0.079	0.124	—
(−)-Cyperene	002387-78-2	0.063	0.094	—
*β*-Bourbonene	005208-59-3	0.146	0.319	—
*β*-Ylangene	020479-06-5	0.157	0.196	—
*β*-Caryophyllene	000087-44-5	7.495	20.022	13.687
*α*-Humulene	006753-98-6	2.236	2.339	—
*a*-Cyperene	002387-78-2	0.283	—	—
Alloaromadendrene	025246-27-9	0.139	0.18	—
Germacrene D	037839-63-7	0.548	1.535	—
*β*-Selinene	017066-67-0	1.112	1.713	—
Longifolene	000475-20-7	0.359	—	—
*δ*-Cadinene	000483-76-1	—	0.324	—
*trans*-Calamenene	073209-42-4	0.036	—	—
Chamazulene	000529-05-5	0.049	—	—
**Oxygenated sesquiterpenes**		**22.72**	**2.66**	**40.82**
Caryophyllene oxide	001139-30-6	8.713	0.133	21.553
Salvial-4(14)-en-1-one	073809-82-2	—	0.133	—
*α*-Humulene epoxide II	019888-34-7	0.763	0.3	—
Junenol	000472-07-1	0.22	0.128	—
Nerolidol	000142-50-7	0.055	—	—
Spathulenol	006750-60-3	1.508	0.221	2.487
Neointermedeol	005945-72-2	9.652	1.16	16.779
11,11-Dimethyl-4,8-dimethylene-bicyclo[7.2.0]undecan-3-ol	079580-01-1	0.304	0.06	—
10,10-Dimethyl-2,6-dimethylene-bicyclo[7.2.0]undecan-5.*beta*.-ol	019431-80-2	1.302	0.443	—
Costol	000515-20-8	0.06	0.08	—
Phytol (3,7,11,15-Tetramethyl-2-hexadecen-1-ol)	000150-86-7	0.14	—	—
**Others**		**2.88**	**2.51**	**0**
*cis*-Sabinyl acetate	139757-62-3	0.508	0.41	—
*iso*-Thujol	007712-79-0	0.734	0.545	—
Bornyl isovalerate	000076-50-6	0.652	0.596	—
Bornyl tiglate	000076-49-3	0.404	0.38	—
2-Methoxy-3-(2-propen-1-yl)phenol,	001941-12-4	0.209	0.433	—
Palmitic acid	000057-10-3	0.373	0.148	—

**Table 3:** Inhibition zones (diameter, mm) of *Artemisia argyi* essential oils against various microorganisms.

Microorganisms	Extraction Method		
	Hydrodistillation	Subcritical Extraction	SDE
*Staphylococcus aureus*	18.23 ^a^ ± 0.26	8 ^g^ ± 0.19	13.23 ^cd^ ± 0.37
*Bacillus subtilis*	17.43 ^b^ ± 0.45	21.88 ^a^ ± 0.39	15.15 ^b^ ± 0.44
*Listeria monocytogenes*	13.2 8^c^ ± 0.51	8.3 ^g^ ± 0.58	13.43 ^c^ ± 0.61
*Escherichia coli*	10.03 ^d^ ± 0.38	9.7 ^e^ ± 0.41	14.78 ^b^ ± 0.46
*Proteus vulgaris*	9.35 ^e^ ± 0.66	10.63 ^d^ ± 0.58	15.8 ^a^ ± 0.46
*Salmonella enteritidis*	10.48 ^d^ ± 0.54	8.93 ^f^ ± 0.66	13.95 ^c^ ± 0.39
*Saccharomyces cerevisiae*	13.53 ^c^ ± 0.58	14.7 ^b^ ± 0.52	11.23 ^f^ ± 0.49
*Aspergillus niger*	10.45 ^d^ ± 0.61	11.3 ^c^ ± 0.53	12.53 ^e^ ± 0.46

Data are average of a duplicate test (mean ± SD) with at least three determinations. ^a,b,c,d,e,f,g^: Values with different superscripts are significantly different in line (*p* < 0.5). Negative control inhibition diameter: not detected.

**Table 4:** MICs (μL/mL) of *Artemisia argyi* essential oils against various microorganisms.

Microorganisms	Extraction Method		
	Hydrodistillation	Subcritical Extraction	SDE
*Staphylococcus aureus*	6.25 ^c^ ± 0.38	12.5 ^b^ ± 0.37	12.5 ^a^ ± 0.37
*Bacillus subtilis*	6.25 ^c^ ± 0.52	3.13 ^d^ ± 0.45	12.5 ^a^ ± 0.42
*Listeria monocytogenes*	6.25 ^c^ ± 0.37	25 ^a^ ± 0.41	6.25 ^b^ ± 0.53
*Escherichia coli*	12.5 ^b^ ± 0.39	12.5 ^b^ ± 0.48	6.25 ^b^ ± 0.51
*Proteus vulgaris*	25 ^a^ ± 0.43	12.5 ^b^ ± 0.37	6.25 ^b^ ± 0.41
*Salmonella enteritidis*	12.5 ^b^ ± 0.42	25 ^a^ ± 0.34	6.25 ^b^ ± 0.52
*Saccharomyces cerevisiae*	6.25 ^c^ ± 0.45	6.25 ^c^ ± 0.4	12.5 ^a^ ± 0.37
*Aspergillus niger*	12.5 ^b^ ± 0.66	12.5 ^b^ ± 0.51	6.25 ^b^ ± 0.36

Data are average of a duplicate (mean ± SD). ^a,b,c^: Values with different superscripts are significantly different in line (*p* < 0.5).
